# Establishment of the Genetic Transformation System of *Trichoderma longibrachiatum* 40418 and Its Induced Resistance Against *Meloidogyne incognita* in Tomato

**DOI:** 10.3390/jof12060422

**Published:** 2026-06-10

**Authors:** Ning Luo, Cailan Wang, Huixuan Yin, Wei Guo, Huixia Li, Yang Jiao

**Affiliations:** 1 College of Plant Protection, Gansu Agricultural University, Lanzhou 730070, China; 18894313158@163.com (N.L.); 18419068535@163.com (C.W.); 18993559261@163.com (H.Y.); guow@gsau.edu.cn (W.G.); 2 School of Resources and Environment, Henan Institute of Science and Technology, Xinxiang 653003, China

**Keywords:** *Trichoderma longibrachiatum*, protoplast, root colonization, induced resistance, defense response

## Abstract

*Trichoderma longibrachiatum* is a filamentous fungus that functions as a biocontrol agent against multiple plant diseases. However, the lack of a genetic transformation system has hindered studies on gene function and the underlying biocontrol mechanisms in this species. In this study, protoplast preparation conditions were optimized using single-factor experiments and response surface methodology, yielding 2.05 × 10^7^ protoplasts/g. PEG-mediated transformation yielded a GFP-labeled strain (GFP-40418). This strain enhanced resistance against *Meloidogyne incognita*, with root galling decreased by 47.6%. Treatment with GFP-40418 enhanced the activities of antioxidant enzymes, including PAL, POX, SOD, PPO, APX, and CAT. Plants treated with GFP-40418 alone showed the highest expression value, with relative transcriptional levels of *PR2*, *Pal1*, *LOX*, *MYC2*, *ETR1*, and *ACO1* that were 29.62, 5.76, 7.30, 3.64, 4.79, 9.99-fold higher in comparison with control plants, respectively. These findings provide a genetic platform for exploring the gene functions of *T. longibrachiatum* 40418 and validate its biocontrol prospect against root-knot nematodes.

## 1. Introduction

*Trichoderma* spp. is a biocontrol fungus widely distributed in the plant root ecosystems. It suppresses many plant pathogens through nutrient competition, mycoparasitism, the production of antimicrobial metabolites, induced resistance, growth promotion, and antibiosis [1,2]. Multiple research findings have demonstrated that *Trichoderma* can exert broad-spectrum inhibitory effects on phytopathogens, including *Botrytis cinerea* [3], *Plasmopara viticola* [4], *Fusarium culmorum* [5], *Phytophthora infestans* [6] and plant-parasitic nematodes [7,8]. Root-knot nematodes, particularly *Meloidogyne incognita*, cause severe yield losses in a wide range of crops worldwide. Chemical nematicides are effective but often associated with environmental contamination and resistance development. Therefore, environmentally friendly biological control strategies have gained increasing attention. The acidic substances produced by *Trichoderma* can dissolve the insoluble trace elements in the soil, providing plants with more nutrients [9]. Our previous studies indicated that *T. longibrachiatum* 40418 has biocontrol potential against *M. incognita* [10]. Although strain 40418 has shown promising activity against M. incognita, the underlying biological basis of its interaction with host plants and nematodes remains unclear.

Genetic transformation systems are indispensable tools for investigating gene function and elucidating the biological processes in biocontrol fungi [11]. However, due to the complex cell wall composition and structural diversity among filamentous fungi, no universal transformation protocol is applicable across different species [12]. In recent years, transformation systems have been successfully established in several filamentous fungi, including *T. reesei* [13], *T. harzianum* [14], *Lecanicillium lecanii* [15], *Acremonium implicatum* [16], and *Colletotrichum falcatum* [17], providing valuable platforms for functional genomics and strain improvement. Several transformation approaches have been developed for filamentous fungi, including polyethylene glycol (PEG)-mediated protoplast transformation [18], *Agrobacterium tumefaciens*-mediated transformation (ATMT) [19], shock wave-mediated transformation [20], electroporation [21,22], and restriction enzyme-mediated integration (REMI) [23]. Among these, the PEG-mediated protoplast transformation technique is widely used due to its operational simplicity and relatively high transformation efficiency [24]. Since protoplast transformation efficiency largely depends on the quality and handling of protoplasts, the optimization of protoplast preparation is a critical prerequisite for establishing an efficient transformation system.

Colonization by *Trichoderma* can promote plant growth and induce plant resistance. Induced resistance has attracted increasing attention as an environmentally friendly and sustainable strategy for managing plant pathogens [1]. Several studies have demonstrated that *Trichoderma* priming can activate multiple defense pathways regulated by signaling molecules [25,26]. For example, the treatment of tomato plants with *T. pubescens* significantly enhanced the activities of defense enzymes (POX, SOD, PPO, and CAT), and increased the expression of related genes (*PAL*, *CHS*, and *HQT*) [27]. Both *T. harzianum* and *T. virens* activate salicylic acid (SA)- and jasmonic acid (JA)-dependent systemic resistance in tomato, with the former conferring protection against *M. incognita*, and the latter enhancing tolerance to *F. oxysporum* [28,29]. These studies support the potential of *Trichoderma* as a biological control agent. Our previous study showed that *Arabidopsis thaliana* treated with *T. longibrachiatum* 40418 exhibited increased callose deposition and ROS (H_2_O_2_) accumulation, and showed enhanced resistance to *Pseudomonas syringae* pv. *tomato* DC3000 and *M. incognita* infection [10]. However, whether *T. longibrachiatum* 40418 is associated with similar defense signaling responses in tomato remains unclear.

In this study, we established a genetic transformation system for *T. longibrachiatum* 40418 and generated a GFP (green fluorescent protein)-labeled strain to visualize fungal colonization of tomato roots. A split-root system was employed to evaluate whether GFP-40418 was associated with the enhanced tomato defense responses and reduced root gall formation caused by *M. incognita*. To further investigate defense-associated responses triggered by strain 40418, the activities of defense-related enzymes and the expression levels of marker genes related to SA, JA, and ET signaling pathways were analyzed.

## 2. Materials and Methods

### 2.1. Strain and Culture Conditions

*T. longibrachiatum* 40418 (CGMCC3.25690) was isolated, identified, and preserved by the Disease Group Laboratory of the Institute of Vegetable and Flower Research, Chinese Academy of Agricultural Sciences. *T. longibrachiatum* 40418 and strain GFP-40418 were cultured on potato dextrose agar (PDA) medium (1 L: 200.0 g potato, 20.0 g glucose, and 18.0 g agar) at 28 °C. Potato dextrose broth (PDB) medium (1 L: 24.0 g PDB powder, Becton, Dickinson and Company, Franklin Lakes, NJ, USA) was used for culturing *T. longibrachiatum* 40418 and strain GFP-40418 for fresh mycelia collection and DNA extraction. T-Top medium (1 L: 200.0 g sucrose, 20.0 g glucose, 10.0 g agar, 2.0 g NaNO_3_, 1.0 g KH_2_PO_4_, 0.5 g MgSO_4_·7H_2_O, and 0.5 g KCl) was used for protoplast regeneration. Bacterial cultures containing plasmid pCH-sGFP were grown in Luria–Bertani (1 L: 5.0 g yeast extract, 10.0 g NaCl, 10.0 g peptone, and 20.0 g agar) medium supplemented with the relevant antibiotic.

### 2.2. Extraction of DNA and RNA

The total genomic DNA of strain GFP-40418 (Mycelia collected from two Petri dishes) was extracted using the Fungal Genomic DNA Rapid Extraction Kit (Biotech, Shanghai, China) following the manufacturer’s protocol. Total RNA of tomato was extracted using the FastPure Plant RNA Kit (TianGen, Beijing, China) and reverse-transcribed with TIAN Script II RT kit (TianGen, Beijing, China).

### 2.3. Preparation of Protoplasts

The cell-wall composition of filamentous fungi is relatively complex, with distinct differences among fungal species. Consequently, the conditions for protoplast preparation vary, including factors such as the type of lytic enzyme, osmotic stabilizer, mycelial age, the enzymatic hydrolysis time, and the enzymatic temperature [30].

Osmotic stabilizing agents: 0.5 mol/L NaCl (A), 0.7 mol/L NaCl (B), 0.6 mol/L glucose (C), 0.8 mol/L glucose (D), 0.6 mol/L KCl (E), 0.8 mol/L KCl (F), 0.6 mol/L mannitol (G), 0.8 mol/L mannitol (H). Types of lytic enzymes: cellulase (A), snailase (B), lysing enzymes (C), driselase (D). Each enzyme was used at a concentration of 20 mg/mL in seven different combinations: A, B, C, D, A + B, A + C, and B + C. Mycelial age (10, 12, 14, 16, and 18 h). Incubation time (1, 2, 3, 4, and 5 h). Incubation temperature (24, 26, 28, 30, and 32 °C). Rotations per minute was tested at 120, 140, 160, 180, and 200 rpm. The yield of protoplasts isolated from 1 g of fresh mycelia under each treatment condition was determined by hemocytometer counting.

### 2.4. Optimization of Protoplast Preparation Conditions for Strain 40418 Using Response Surface Methodology

Based on the single-factor experimental results, three factors and their corresponding levels were chosen for optimization. The detailed experimental conditions are provided in Supplementary Table S1. Subsequently, a Box–Behnken design comprising 17 experimental runs was established using Design-Expert 13 software. The Box–Behnken design is shown in Supplementary Table S2. Each condition was tested in triplicate.

### 2.5. PEG-Mediated Transformation

The transformation workflow was modified from the method of *T. hamatum* [11] and *Purpureocillium lilacinum* [31]. Aurintricarboxylic acid (1 µmol) and pCH-sGFP plasmid (5.0 µg) were added to a centrifuge tube, and the volume was adjusted to 60 µL with TEC buffer (200 mL: 0.3152 g Tris-HCl, 0.0585 g EDTA and 1.1762 g CaCl_2_·2H_2_O). The mixture was incubated on ice for 20 min. The mixture was centrifuged at 12,000 rpm for 2 min at 4 °C, and the supernatant was collected. Protoplast suspension (100 µL) was added to the supernatant and incubated on ice for 20 min. Then, 160 µL of 60% PEG 4000 (200 mL:120.0 g PEG 4000, 25.1 g MOPS) was added, and the mixture was incubated at room temperature for 15 min. Next, 1 mL of STC buffer was added to the sample and mixed gently. The mixture was then centrifuged at 4000 rpm for 5 min at 4 °C. After centrifugation, the supernatant was carefully discarded, and the pellet was resuspended in 200 μL STC buffer. Finally, the samples were mixed with 25 mL of T-Top medium. The mixture was evenly spread onto five PDA plates and incubated at 28 °C for 13–15 h. Then, 10 mL of screening T-Top medium (containing hygromycin B) was added to each Petri dish. After incubating at 28 °C for 1–2 d, transformant growth was monitored daily, and colonies were transferred to fresh PDA plates containing hygromycin B.

### 2.6. Identification of Transformants

Putative transformants were transferred to PDA plates supplemented with hygromycin B using sterile toothpicks. Mycelia from the transformants were mounted onto glass slides and examined for green fluorescence under a fluorescence microscope (Olympus IX53, Tokyo, Japan). Putative GFP-positive transformants were collected after 15–20 h of incubation for genomic DNA extraction. Transformants were identified by PCR, and primer information is provided in Supplementary Table S3. Single spores were isolated from transformants showing green fluorescence and subcultured on PDA plates for six generations. Changes in GFP fluorescence were observed during subculturing. A transformant that maintained detectable GFP fluorescence and showed growth comparable to the wild-type strain was selected and designated GFP-40418 for subsequent analyses.

### 2.7. Measurement of Transformed Strain Growth Rate

The wild-type strain 40418 and GFP-40418 were cultured at 28 °C. Colony diameters were measured at 6, 12, and 24 h, and growth rates were then calculated. Ten biological replicates were included for each strain. Differences in growth rates were analyzed by one-way ANOVA, followed by Duncan’s multiple-range test using SPSS software (*p* < 0.05).

### 2.8. Induced Resistance Assays

Tomato (*Solanum lycopersicum* cv. Lichun) plants were used to evaluate the effect of GFP-40418 on induced resistance. We adopted the split-root approach, as depicted in Figure S1. The assay included three treatments: GFP-40418, wild-type 40418, and a water control (CK). Conidial suspension of GFP-40418 and wild-type 40418 was adjusted to 3.0 × 10^8^ conidia/mL with sterile distilled water and used for soil inoculation. Each plant was inoculated with 1 mL of the conidial suspension. Seven days later, 600 s-stage juveniles (J2s) of *M. incognita* were inoculated into the soil on the opposite side of the split-root system. Root gall formation was assessed at 40 days post-inoculation (dpi). Each biological replicate consisted of 20 individual plants. Root galls with a diameter > 1 mm were counted under a stereomicroscope to ensure consistent counting standards across all samples. The experimental procedures were conducted with reference to the methods in our previous research [10,11].

### 2.9. Measurement of Defense-Related Enzyme Activities

Four-week-old tomato plants were treated with a conidial suspension of GFP-40418. Root tissues were collected at 0, 3, 6, 9, 12, 15, and 18 dpi and immediately frozen in liquid nitrogen. Subsequently, the resulting homogenate was used to measure the activities of defensive enzymes, including phenylalanine ammonia lyase (PAL), catalase (CAT), superoxide dismutase (SOD), peroxidase (POD), ascorbate peroxidase (APX), and polyphenol oxidase (PPO) [27,32].

### 2.10. qRT-PCR Analysis

Sampling time and methods were consistent with those described above. The expression levels of defense-related genes, including *PR2*, *Pal1*, *LOX*, *MYC2*, *ETR1*, and *ACO1*, were quantified by qRT-PCR. Primer sequences are listed in Supplementary Table S4. *Aactin* was used as the internal reference gene. qRT-PCR was performed using a QuantStudio 5 Real-Time PCR System (Thermo Fisher Scientific, Waltham, MA, USA) Reactions were set up with ChamQ Blue Universal SYBR qPCR Master Mix (Vazyme, Cat. No. Q312, Nanjing, China) in strict accordance with the manufacturer’s guidelines. Three biological replicates were included for each treatment, and each biological replicate was analyzed with three technical replicates. Statistical analyses were performed using ΔCt values, while relative expression levels were calculated using the 2^−ΔΔCt^ method for presentation [33].

### 2.11. Statistical Analysis

Statistical analysis was performed using Microsoft Excel 2010 and SPSS version 27 (IBM, Armonk, NY, USA). Significant differences were evaluated by Duncan’s multiple-range test following one-way ANOVA (*p* < 0.05). All experiments were performed in more than three independent replicates, and data are presented as mean ± standard deviation (SD).

## 3. Results

### 3.1. Effects of Different Experimental Factors on Protoplast Yield

**Figure 1:**
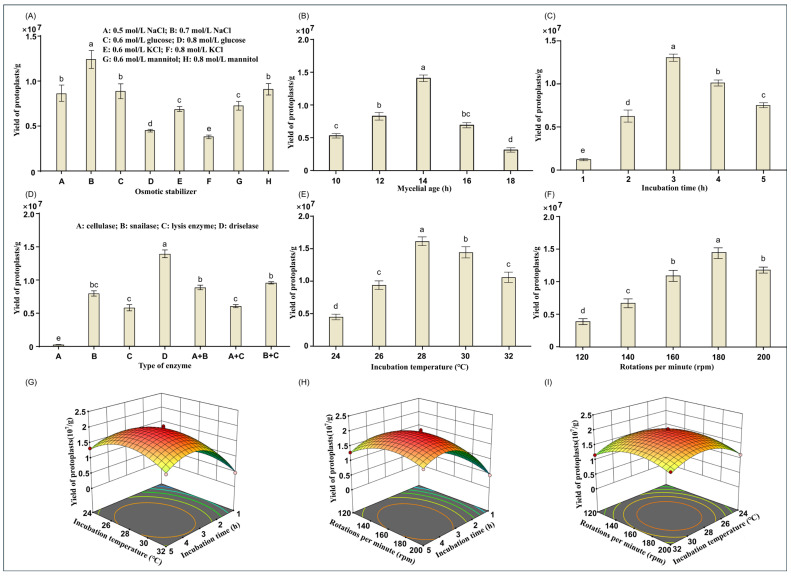
Optimization of protoplast preparation conditions for *T. longibrachiatum* 40418. (**A**–**F**) Effects of different experimental factors on protoplast yield of *T. longibrachiatum* 40418. (**G**–**I**) Response surface plots for the optimization of protoplast preparation conditions. (**A**) Osmotic stabilizer. (**B**) Mycelial age. (**C**) Incubation time. (**D**) Type of enzyme. (**E**) Incubation temperature. (**F**) Rotational speed. (**G**) Incubation time and incubation temperature. (**H**) Rotations per minute and incubation time. (**I**) Rotations per minute and incubation temperature. The different letters indicate the significant difference, determined by one-way ANOVA followed by the Duncan’s multiple-range test (*p* < 0.05).

Strain 40418 was identified as *T. longibrachiatum*, based on the *ITS*, *tef1α*, and *rpb2* sequences analyses. Hygromycin B at 50 μg/mL was selected for transformant screening. However, systematic research on the genetic system conditions for strain 40418 remains lacking [10]. The highest protoplast yield (1.25 × 10^7^ protoplasts/g) was achieved when 0.7 mol/L NaCl was used as the osmotic stabilizer, significantly outperforming all other treatments (Figure 1A). Cell wall-degrading enzymes are indispensable for protoplast preparation. Driselase showed the highest enzymatic efficiency in this experiment, yielding 1.39 × 10^7^ protoplasts/g, significantly higher than the results obtained with cellulase, snailase and lysis enzymes (*p* < 0.05). Cellulase showed the poorest performance, with a yield of only 2.83 × 10^5^ protoplasts/g (Figure 1D). The highest protoplast yield was obtained using 14 h-old mycelia, and this yield (1.43 × 10^7^ protoplasts/g) was significantly higher than those of the other treatments (Figure 1B). Both enzymatic digestion time and temperature affected protoplast yield (Figure 1C,E). The highest protoplast yield (1.43 × 10^7^ protoplasts/g) was observed at 180 rpm (Figure 1F).

### 3.2. Condition Optimization via Box–Behnken Design

Based on the results of preliminary single-factor experiments, a Box–Behnken design was employed, with incubation time (A), incubation temperature (B), and rotations per minute (C) as independent variables, and protoplast yield as the response variable (Table S1). Additionally, the interactive effects of these factors on the response variable were investigated, and the experimental results were summarized in Table 1. The above results were subsequently analyzed using Design-Expert software, yielding the following second-order polynomial regression equation:

Y = −28.06537 + 1.35037A + 1.55369B + 0.066144C − 0.007187AB + 0.00075AC + 0.000141BC − 0.169750A^2^ − 0.028281B^2^ − 0.000218C^2^

ANOVA-based statistical validation demonstrated that the model was highly significant (F = 210.21, *p* < 0.0001) and exhibited strong predictive capability, with an adjusted R^2^ of 0.9916, indicating good model fit. The nonsignificant lack-of-fit test (*p* = 0.5480) indicated that the model adequately fitted the experimental data (Table 2). Main effects analysis indicated that incubation time (A, *p* < 0.01), incubation temperature (B, *p* < 0.01), rotations per minute (C, *p* < 0.01) and the quadratic terms (A^2^, B^2^, and C^2^; *p* < 0.01) were key determinants of protoplast yield. Three-dimensional response surface analysis revealed that interaction effects followed the hierarchy AC > AB > BC, as evidenced by contour gradient steepness (Figure 1G–I). Model optimization predicted a maximum yield of 2.05 × 10^7^ protoplasts/g at 167.43 rpm, 3.70 h, and 27.95 °C. Experimental validation under slightly adjusted conditions (170 rpm, 4.0 h, 28.0 °C) achieved 1.89 × 10^7^ protoplasts/g, representing 92.2% agreement with the predicted value. The predicted and experimental values showed good agreement. These results supported the practical feasibility of using 170 rpm, 4.0 h, and 28.0 °C as the validated conditions for protoplast preparation.

In the single-factor experiment, the highest protoplast yield was observed at 180 rpm (Figure 1F). After response surface methodology (RSM)optimization and validation, 170 rpm was selected as the practical optimized rotations per minute. In summary, based on the single-factor experiments and RSM validation, the practical optimized conditions for preparing *T. longibrachiatum* 40418 protoplasts were 0.7 mol/L NaCl as the osmotic stabilizer, 14 h mycelia, and enzymatic digestion with 20 mg/mL driselase at 28 °C and 170 rpm for 4 h.

### 3.3. Identification of GFP-Tagged Transformants

**Figure 2:**
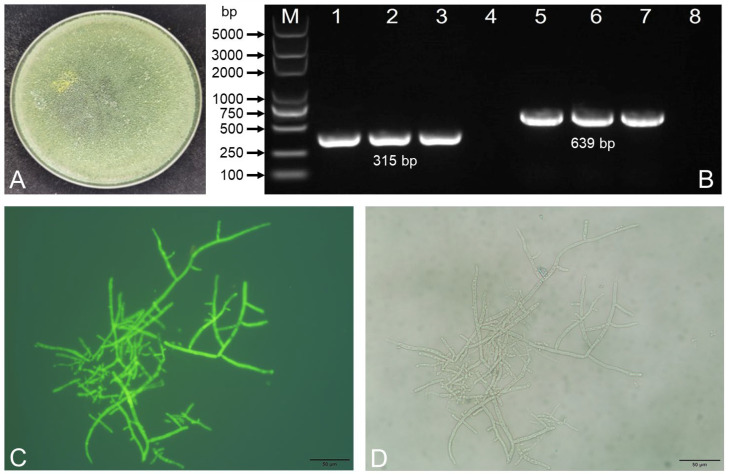
Identification and phenotypic characterization of GFP-labeled transformants. (**A**) Colony morphology of GFP-40418. (**B**) Agarose gel electrophoresis of PCR products for screening the *GFP* and *Hyg* gene in transformants. M: DL2000 Marker; Lanes 1 to 3: PCR amplification of the GFP fragment from transformants; Lane 4: control; Lanes 5 to 7: The size of the *Hyg* gene fragment in transformants; Lane 8: control. (**C**,**D**) Fluorescent images and bright-field images of the transformant GFP-40418. All scale bars represent 50 μm.

Protoplasts of strain 40418 were prepared using optimized conditions. The pCH-sGFP plasmid was introduced into protoplasts via PEG-mediated transformation. A total of 36 transformants were obtained. Green fluorescence was detected in 34 transformants, and most GFP-positive transformants showed similar colony morphology. Three transformants were then randomly chosen for molecular characterization. After a 7 d culture on PDA, the morphology of the transformants, including colony morphology and colony color, was similar to that of the wild-type strain 40418 (Figure 2A). Three transformants were randomly selected, and genomic DNA was extracted. The GFP-f/GFP-r primers amplified a 315 bp fragment (lanes 1 to 3) from the transformants and positive control (pCH-sGFP), but no amplification was detected from wild-type strain 40418 (lane 4) (Figure 2B). The Hyg-f/Hyg-r primers amplified 639 bp fragment (lanes 5 to 7) from the transformants and positive control (pCH-sGFP), but no amplification was detected from wild type strain 40418 (lane 8) (Figure 2B), indicating the pCH-sGFP plasmid was effectively transferred into wild-type strain 40418. The transformant mycelium and conidia exhibited green fluorescence under the microscope, indicating successful GFP expression in the transformants (Figure 2C,D). At 24 h, transformants exhibited a marginally higher mycelial growth rate than the wild-type strain (2.32 ± 0.02 mm/h) (Table S5). GFP-40418 (2.34 ± 0.02 mm/h) exhibited nonsignificant differences (*p* > 0.05) from the wild-type strain 40418. Transformants exhibited comparable growth rates to the wild-type strain 40418, suggesting that GFP labeling did not substantially affect colony morphology or mycelial growth under the tested conditions (Table S5). Therefore, GFP-40418 was selected as a representative transformant for subsequent experiments because its colony morphology and growth rate were comparable to those of the wild-type strain.

### 3.4. Biocontrol Effect of Transformants

**Figure 3:**
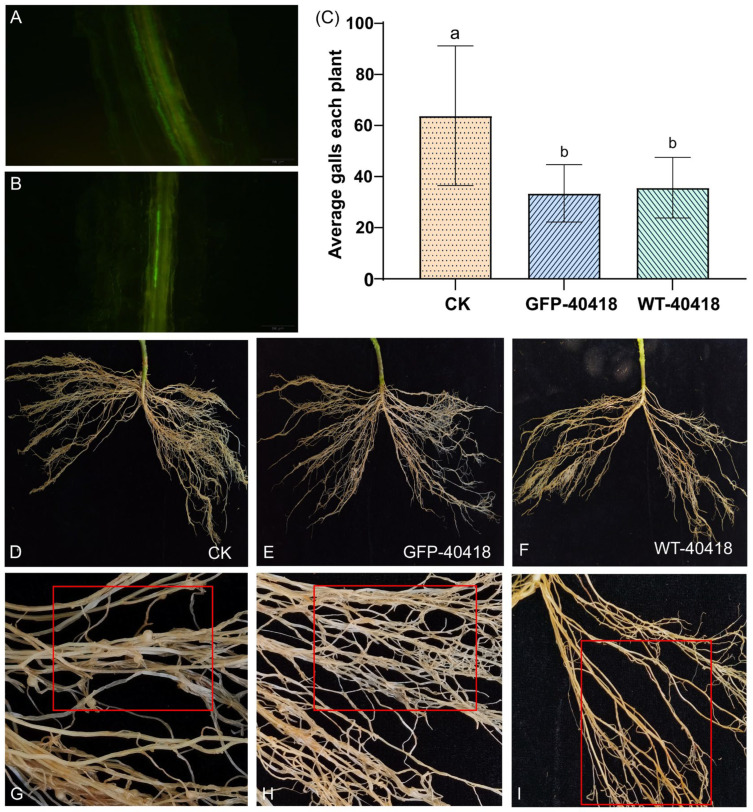
Assessment of root colonization capacity and biocontrol efficacy of GFP-40418. (**A**,**B**) Observation of colonization in tomato roots. (**C**) Average number of root galls in tomato plants treated with GFP-40418 and WT-40418 at 40 dpi. The bar chart represents the mean values ± SD. (**D**–**I**) The tomato root phenotypes of GFP-40418-treated plants at 40 dpi. Lower panels show enlarged views of the corresponding upper panels. An equivalent volume of water served as the control. Different letters (a and b) indicate significant differences according to one-way ANOVA followed by Duncan’s multiple-range test (*p* < 0.05). All the experiments were independently repeated thrice, showing similar results.

Among the three transformants, GFP-40418 maintained detectable GFP fluorescence under the tested conditions and showed the greatest similarity to the wild-type strain 40418 in morphology and growth (Table S5). The GFP-40418 strain exhibited strong green fluorescence after six successive rounds of subculture under hygromycin selection, supporting its application for subsequent tomato root colonization analysis. Accordingly, this transformant was selected for further analyses. Hyphal attachment to the surface of tomato roots was observed at 4 dpi (Figure 3A). By 7 dpi, the hyphae had extended longitudinally along root tissues parallel to the root axis (Figure 3B). These observations provided qualitative visual evidence that GFP-40418 could colonize tomato roots. To investigate whether the GFP-40418 strain can induce plant resistance, a split-root experiment was conducted. Our results indicated that GFP-40418 treatment reduced root gall formation compared with the water control. (Figure 3C–G). Compared with the water control, GFP-40418 treatment reduced the number of root galls by 47.6%. (Figure 3C). However, the underlying control mechanism remains to be further elucidated. Only one GFP-labeled transformant was used in the biological assays, which represents a limitation of this study.

### 3.5. Defense-Related Enzyme Activities

**Figure 4:**
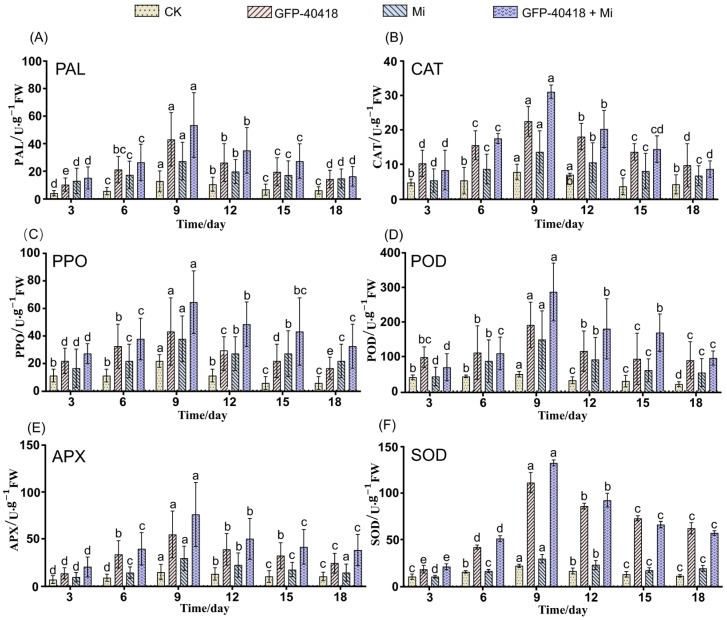
Assay of defense-related enzyme activities. (**A**) Phenylalanine ammonia lyase (PAL). (**B**) Catalase (CAT). (**C**) Polyphenol oxidase (PPO). (**D**) Peroxidase (POD). (**E**) Ascorbate peroxidase (APX). (**F**) Superoxide dismutase (SOD). Root samples were collected at 0, 3, 6, 9, 12, 15 and 18 dpi. The bar chart represents the mean values ± SD. Significant differences are represented by the various letters (a–e) at *p*-value ≤ 0.05. Data are presented as the mean ± SD of three biological replicates.

To further investigate whether GFP-40418 was associated with changes in tomato defense responses, the activities of six defense-related enzymes were measured. GFP-40418 treatment was associated with increased activities of several defense-related enzymes in tomato plants, as recorded in group treatments GFP-40418 and GFP-40418 + Mi. (Figure 4). The activities of all tested enzymes initially increased and then decreased, reaching their peak at 9 dpi. The GFP-40418+Mi treatment exhibited the highest PAL activity value (53.56 U/g FW), followed by GFP-40418-treated plants (43.11 U/g FW) (Figure 4A). CAT activity was elevated in response to *M. incognita* infection in plants treated with GFP-40418 (Figure 4B). Compared with control, infected plants treated with GFP-40418 exhibited the highest level of CAT activity, followed by plants treated with GFP-40418. PPO activity peaked at 9 dpi and decreased gradually thereafter, and was significantly higher in treated versus control plants (Figure 4C). Peroxidase (POD) activity in control tomato plants remained relatively stable. POD activity peaked across all treatments at 9 dpi, with the highest value detected in the GFP-40418 + Mi treatment (286.67 U/g FW) (Figure 4D). APX activity in plants inoculated with *M. incognita* alone was lower than that in plants treated with GFP-40418 alone and in the GFP-40418 + Mi treatment (Figure 4E). Similarly, SOD activity was significantly increased in plants treated with GFP-40418 alone. The GFP-40418 + Mi treatment showed the highest SOD activity, followed by GFP-40418 treatment (132.52 and 111.33 U/g FW, respectively) (Figure 4F).

Two-way ANOVA showed that treatment and time significantly affected PAL, CAT, PPO, POD, SOD, and APX activities. The treatment × time interaction was significant only for SOD activity (Table S6).

### 3.6. Relative Gene Expression Levels

**Figure 5:**
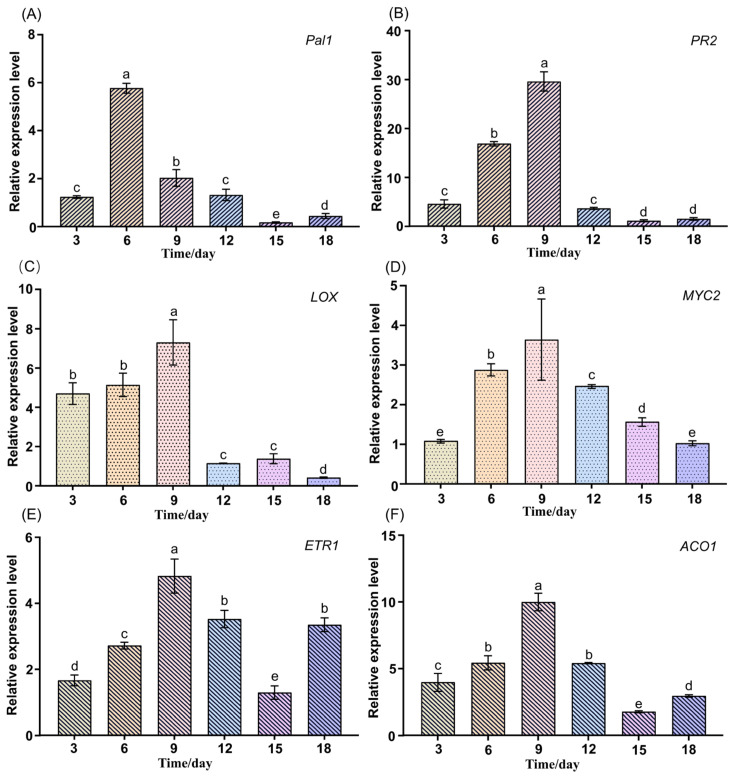
Relative expression levels of *PR2*, *Pal1*, *LOX*, *MYC2*, *ETR1*, and *ACO1* genes in tomatoes treated with GFP-40418. (**A**,**B**) Marker genes *PR2* and *Pal1* of the SA signaling pathway. (**C**,**D**) Marker genes *LOX* and *MYC2* of the JA signaling pathway. (**E**,**F**) Marker genes *ETR1* and *ACO1* of the ET signaling pathway. Sampling was performed at 0, 3, 6, 9, 12, 15 and 18 dpi. The bar chart represents the mean values ± SD. Different letters indicate significant differences according to one-way ANOVA followed by Duncan’s multiple-range test (*p* < 0.05). Expression levels were normalized to *Actin* and calibrated against the untreated control at each corresponding time point.

The expression levels of six defense-related genes (*PR2*, *Pal1*, *LOX*, *MYC2*, *ETR1*, and *ACO1*) were analyzed to investigate whether GFP-40418 treatment was associated with activation of tomato defense responses. *PR2* and *Pal1*, which are commonly used as marker genes of the SA signaling pathway, were analyzed by qRT-PCR. Results of the gene expression analysis are shown in Figure 5. *PR2* expression peaked at 9 dpi, with a 29.62-fold increase (Figure 5A), whereas *Pal1* expression peaked at 6 dpi, with a 5.80-fold increase (Figure 5B). *MYC2* and *LOX* serve as marker genes in the JA signaling pathway, and their expression profiles are presented in Figure 5. The transcript levels of both *MYC2* and *LOX* peaked concurrently at 9 dpi, with 3.63-fold and 7.30-fold increases, respectively (Figure 5C,D). Likewise, *ETR1* and *ACO1* (ethylene pathway genes) displayed similar expression patterns, with 4.79- and 9.99-fold upregulation at 9 dpi, respectively (Figure 5E,F). Overall, inoculation with strain GFP-40418 significantly upregulated the expression of marker genes associated with the SA, JA, and ethylene (ET)signaling pathways in tomato, suggesting that these three defense signaling pathways may be involved in plant disease resistance responses.

## 4. Discussion

Many crops are frequently affected by *M. incognita*, which impairs root growth and reduces crop yields, leading to substantial economic losses [34,35]. Nematicides are widely used as chemical control agents and effectively manage *M. incognita* [36]. However, the extensive use of chemical nematicides raises concerns regarding environmental safety, residues, and resistance risks. Many microorganisms are used as biocontrol agents, serving as a safe, sustainable, and environmentally friendly alternative to chemical pesticides [37,38]. Previous studies have reported that several biocontrol microorganisms suppress plant-parasitic nematodes, including *P*. *lilacinum* [39], *T. asperellum* [40], and *T. harzianum* [41]. In this study, GFP-40418 showed qualitative visual evidence of tomato root colonization and was associated with enhanced defense responses and reduced root gall formation, with a 47.6% reduction compared with the control (Figure 3), the current efficacy assessment relies solely on root gall phenotyping. Quantification of egg masses, eggs per gram of root, nematode reproduction factor, and plant growth performance is therefore indispensable to fully and quantitatively characterize the strain’s biocontrol potential against *M*. *incognita*. Therefore, understanding the interaction between strain 40418, tomato roots, and nematodes is important for further development of biological control strategies.

In recent years, microbial agents have received increasing attention in biocontrol research [42]. Currently, the biocontrol mechanisms of *T. longibrachiatum* 40418 are poorly understood, and establishing a genetic system is crucial for further exploring its biocontrol functions. Efficient preparation of fungal protoplasts is essential for facilitating genetic manipulation in filamentous fungi [2]. This study found that the type of osmotic stabilizer, the composition of cell wall-degrading enzymes, incubation time, mycelial age, incubation temperature, and rotations per minutes significantly influenced protoplast yield. Based on single-factor experiments and RSM validation, the practical optimized conditions were established as follows: 14 h logarithmic-phase mycelia were digested with 20 mg/mL driselase in 0.7 M NaCl at 28 °C for 4 h with shaking at 170 rpm. The optimal enzymatic conditions differed from those reported for other filamentous fungi, possibly because of species-specific differences in cell wall composition and mycelial physiology [43]. These differences may be attributed to the inherent characteristics of fungal species. Additionally, this study demonstrated that while enzymatic hydrolysis for 4–5 h yielded a substantial number of protoplasts, a proportion of them underwent rupture, thereby compromising transformation efficiency. To improve preparation efficiency, multivariate optimization was conducted using RSM based on a Box–Behnken design. Mathematical modeling identified optimal enzymatic conditions of 167.43 rpm, 3.70 h, and 27.95 °C, yielding 2.05 × 10^7^ protoplasts/g. Experimental validation under slightly adjusted conditions (170 rpm, 4.0 h, 28.0 °C) produced 1.89 × 10^7^ protoplasts/g, showing 92.2% consistency with the predicted value. Similar optimization trends have also been reported in other filamentous fungi [43]. Furthermore, the established transformation system provides a useful tool for future functional studies and genetic modification of *T. longibrachiatum* 40418 and may provide a reference for transformation studies in related *Trichoderma* species.

*Trichoderma* spp. are capable of protecting plants from pathogens through the induction of plant resistance, and defense-related enzyme activities are commonly used as indicators of induced plant responses [44,45]. Multiple enzymatic activities participate in the complex interactions among *Trichoderma*, plants, and pathogens. Previous studies have shown that *Trichoderma* spp. can enhance rice disease resistance by increasing the activities of core defense-related enzymes, including PAL, PPO, CAT, and POD [46]. Treatment with *T. afroharzianum* significantly enhanced the activities of antioxidant enzymes including CAT, POD, SOD, and PPO, whereas pathogen-infected tomato plants exhibited marked accumulation of the oxidative stress markers MDA and H_2_O_2_ [44]. A key finding of this study is that *T. longibrachiatum* strain 40418 may alleviate nematode-associated damage through induction of plant defense responses. We observed that treatment of tomato plants with GFP-40418 spore suspensions enhanced the activities of defense-related enzymes, including PAL, CAT, PPO, POD, APX, and SOD. PAL is closely associated with phenylpropanoid metabolism and SA-related defense responses, while CAT, PPO, POD, APX, and SOD are involved in ROS metabolism and have been associated with plant defense responses, including PTI- and ISR-related processes [47,48,49,50]. Our results revealed that PAL activity increased by up to 3.4-fold compared with the control at 9 dpi after treatment with *T. longibrachiatum* (Figure 4A). The results were consistent with other studies [46,51]. Similarly, Lv et al. reported that CAT, SOD, POD, and PPO activities were significantly increased compared with the control in all treatments after inoculation of *Monilia yunnanensis* with *T. longibrachiatum* T6 [52]. Antagonistic *Bacillus* spp. significantly enhanced antioxidant defense enzyme activities in rice leaves and roots under hydroponic and soil conditions, respectively, with SOD (1.7–1.9-fold), POD (3.5–4.1-fold), PPO (3.0–3.8-fold), and PAL (3.9–4.4-fold). Similar trends were observed in the present study. These findings suggest that increased activities of PAL, CAT, PPO, APX, SOD, and POD may be associated with GFP-40418-mediated defense responses against *M. incognita* in tomatoes. Further studies are needed to clarify the underlying molecular mechanisms.

*Trichoderma* spp. are widely used to study plant–microbe interactions and induced resistance [53]. *Trichoderma* spp. can produce diverse secondary metabolites, some of which may function as elicitors of plant defense responses. SA, JA, and ET are major signaling molecules involved in plant-induced resistance [54,55]. Earlier studies indicated that *Trichoderma* primes SA-mediated defense responses, thereby restricting nematode root invasion. Subsequently, *T. harzianum* enhances JA-regulated defenses, counteracting nematode-induced suppression of JA-dependent immunity and ultimately reducing gall formation and fecundity [28]. *T. longibrachiatum* induces systemic resistance in cucumber against *B. cinerea* by activating signaling pathways associated with the phytohormones JA/ET and SA [3]. The treatment of tomato roots with *T. asperellum* significantly upregulated the expression levels of JA and ET signaling pathway genes *ETR1* and *LOX1*, thereby enhancing plant resistance to pathogens [56]. Furthermore, several studies reported increased expression of JA/ET signaling-related genes following *Trichoderma* inoculation, as evidenced by the upregulated expression of JA/ET signaling pathway-related genes including *PAL*, *LOX*, *ETR1* and *CTR1* [57,58,59]. In this study, the expression of defense response-related genes (*PR2*, *Pal1*, *LOX*, *MYC2*, *ETR1*, and *ACO1*) was upregulated in tomato plants following inoculation with GFP-40418 (Figure 5). Notably, the *PR2* showed the highest increase, reaching 29.62-fold at 9 dpi (Figure 5A). These results suggest that SA-, JA-, and ET-related defense responses may be associated with the tomato response to GFP-40418. Additional studies are required to clarify the relevant defense signal transduction pathways.

## 5. Conclusions

In this study, we established an optimized protoplast preparation and genetic transformation system for *T. longibrachiatum* 40418, and generated the GFP-labeled transformant, GFP-40418. Single-factor experiments combined with response surface methodology identified the validated practical conditions for protoplast preparation conditions (170 rpm, 4.0 h, 28.0 °C), yielding 1.89 × 10^7^ protoplasts/g, which corresponded to 92.2% agreement with the predicted value. GFP-40418 enhanced defense responses and suppressed root gall formation, reducing gall incidence by 47.6% under controlled conditions. GFP-40418 treatment was also associated with increased expression of marker genes related to SA, JA, and ET defense signaling pathways, including *PR2*, *Pal1*, *LOX*, *MYC2*, *ETR1*, and *ACO1*. Establishment of the genetic transformation system will provide an efficient tool to facilitate future functional studies of *T. longibrachiatum* 40418 and its role in inducing plant defense responses. In addition, our findings provide a basis for future development of *Trichoderma*-based biocontrol strategies against plant-parasitic nematodes.

## Figures and Tables

**Table 1:** The results of Box–Behnken design test.

Test No.	A: Incubation Time (h)	B: Incubation Temperature (°C)	C: Rotations Per Minute (rpm)	D: Protoplast Yield (1 × 10^7^ Protoplasts/g)
1	1	24	160	0.21
2	5	24	160	1.33
3	3	32	120	1.17
4	1	28	120	0.41
5	3	32	200	1.46
6	3	28	160	2.02
7	5	32	160	1.36
8	3	28	160	1.92
9	5	28	120	1.29
10	3	28	160	1.96
11	3	24	120	0.93
12	1	28	200	0.45
13	3	28	160	1.98
14	3	28	160	1.91
15	3	24	200	1.13
16	1	32	160	0.47
17	5	28	200	1.57

**Table 2:** Variance analysis of regression model.

Source	Sum of Squares	df	Mean Square	F-Value	*p*-Value	Significance
Model	5.81	9	0.6458	210.21	<0.0001	**
A	2.01	1	2.01	654.27	<0.0001	**
B	0.0924	1	0.0924	30.09	0.0009	**
C	0.0820	1	0.0820	26.70	0.0013	**
AB	0.0132	1	0.0132	4.30	0.0767	-
AC	0.0144	1	0.0144	4.69	0.0671	-
BC	0.0020	1	0.0020	0.6591	0.4436	-
A^2^	1.94	1	1.94	631.88	<0.0001	**
B^2^	0.8022	1	0.8022	261.13	<0.0001	**
C^2^	0.5128	1	0.5128	166.93	<0.0001	**
Residual	0.0215	7	0.0031			
Lack of fit	0.0134	3	0.0045	2.22	0.5480	not significant
Pure error	0.0081	4	0.0020			
Cor total	5.83	16				

The difference was not significant (*p* > 0.05), ** the difference was very significant (*p* < 0.01); - was no data.

## Data Availability

The original contributions presented in this study are included in the article/Supplementary Materials. Further inquiries can be directed to the corresponding authors.

## Supplementary Materials

The following supporting information can be downloaded at: https://www.mdpi.com/article/10.3390/jof12060422/s1, Figure S1: Schematic drawing of the split-root approach; Figure S2: Gene-specific amplification profile; Table S1: Response surface test design factors and levels; Table S2: Box–Behnken Design experimental design; Table S3: Gene profile and functions; Table S4: List of oligonucleotides used in this study; Table S5: Growth rates of wild-type *T. longibrachiatum* 40418 and GFP-40418; Table S6: Two-way ANOVA analysis of enzyme activity time-course data.
