# Supplementary material for: Establishment of the Genetic Transformation System of Trichoderma longibrachiatum 40418 and Its Induced Resistance Against Meloidogyne incognita in Tomato
Source: J Fungi (Basel). 2026 Jun 10;12(6):422. doi: 10.3390/jof12060422 (PMC13300766; doi:10.3390/jof12060422)
Supplement: Supplementary file 1 [file jof-12-00422-s001.zip › jof-4321515-supplementary.pdf]

Supporting Information for：

Establishment of the Genetic Transformation System of *Trichoderma longibrachiatum* 40418 and Its Induced Resistance Against *Meloidogyne incognita* in Tomato

Table S1. Factors and levels used in the response surface design

Factor	Code	Level		
		-1	0	1
Incubation time (h)	A	1	3	5
Incubation temperature (°C)	B	24	28	32
Rotations per minute (rpm)	C	120	160	200

Table S2. Box-Behnken experimental design

Test No.	A:Incubation time (h)	B: Incubation temperature (°C)	C: Rotations per minute (rpm)	D: Protoplast yield (1×10^7 protoplasts /g)
1	1	24	160	
2	5	24	160	
3	3	32	120	
4	1	28	120	
5	3	32	200	
6	3	28	160	
7	5	32	160	
8	3	28	160	
9	5	28	120	
10	3	28	160	
11	3	24	120	
12	1	28	200	
13	3	28	160	
14	3	28	160	
15	3	24	200	
16	1	32	160	
17	5	28	200	

Table S3. Gene profile and functions

Gene	Full Name	Core Function
PR2	Pathogenesis-Related Protein 2	Antimicrobial defense; cell wall modification
Pal1	Phenylalanine Ammonia-Lyase 1	Phenylpropanoid pathway initiation
LOX	Lipoxygenase	Oxylipin/JA biosynthesis
MYC2	Transcription factor	JA signaling master regulator
ETR1	Ethylene Receptor 1	Ethylene perception
ACO1	1-aminocyclopropane-1-carboxylate oxidase 1	Ethylene biosynthesis

Table S4. List of oligonucleotides used in this study

Primer name	Sequence	Usage
gfp-f	ACTCGCTCTACCTACTTCGG	Identification of transformants
gfp-r	TGTACAGTGACCGGTGACTC	Identification of transformants
hyg-f	ACGAGATCAAGCAGATCAACG	Identification of transformants
hyg-r	GTAGTAGGTTGAGGCCGTTG	Identification of transformants
Actin-f	TGTCCCTATTTACGAGGGTTATGC	Reference
Actin-r	AGTTAAATCACGACCAGCAAGAT	Reference
PR2-f	GGACACCCTTCCGCTACTCTT	qPCR analysis
PR2-r	TGTTCCTGCCCCTCCTTTC	qPCR analysis
LOX-f	ATCTCCCAAGTGAAACACCACA	qPCR analysis
LOX-r	TCATAAACCCTGTCCCATTCTTC	qPCR analysis
ETR1-f	GTTGCCTGCTGACGACTTGC	qPCR analysis
ETR1-r	GCACCGAACTGCACAAGAACC	qPCR analysis
Pal1-f	CGCTATGCTCTCCGAACATCTC	qPCR analysis
Pal1-r	ATTCACCGAGTTAATCTCCCTCTC	qPCR analysis
MYC2-F	GTGCTTCCAGTGCCAATGTG	qPCR analysis
MYC2-R	GCCCGAAGAAGGCAAAACTG	qPCR analysis
ACO1-F	GCGCCACTCTATTGTGGTTA	qPCR analysis
ACO1-R	TGCATCACTTCCTGGATTGT	qPCR analysis

Table S5. Growth rates of wild-type *T. longibrachiatum* 40418 and GFP-40418

Strain	Growth rate (mm/h)		
	6 h	12 h	24 h
40418- WT	2.00±0.04	1.90±0.03	2.32±0.02
GFP-40418	1.98±0.06	1.89±0.02	2.34±0.02
P value(α=0.05)	0.36	0.51	0.43

Table S6. Two-way ANOVA analysis of enzyme activity time-course data.

Parameter	Treatment effect	Time effect	Treatment × Time interaction
PAL activity	$P < 0.001$	$P < 0.001$	$P = 0.746$
CAT activity	$P < 0.001$	$P < 0.001$	$P = 0.680$
PPO activity	$P < 0.001$	$P = 0.005$	$P = 0.989$
POD activity	$P < 0.001$	$P < 0.001$	$P = 0.312$
APX activity	$P < 0.001$	$P < 0.001$	$P = 0.709$
SOD activity	$P < 0.001$	$P < 0.001$	$P < 0.001$

Two-way ANOVA was performed using treatment, time, and treatment × time interaction as fixed factors.

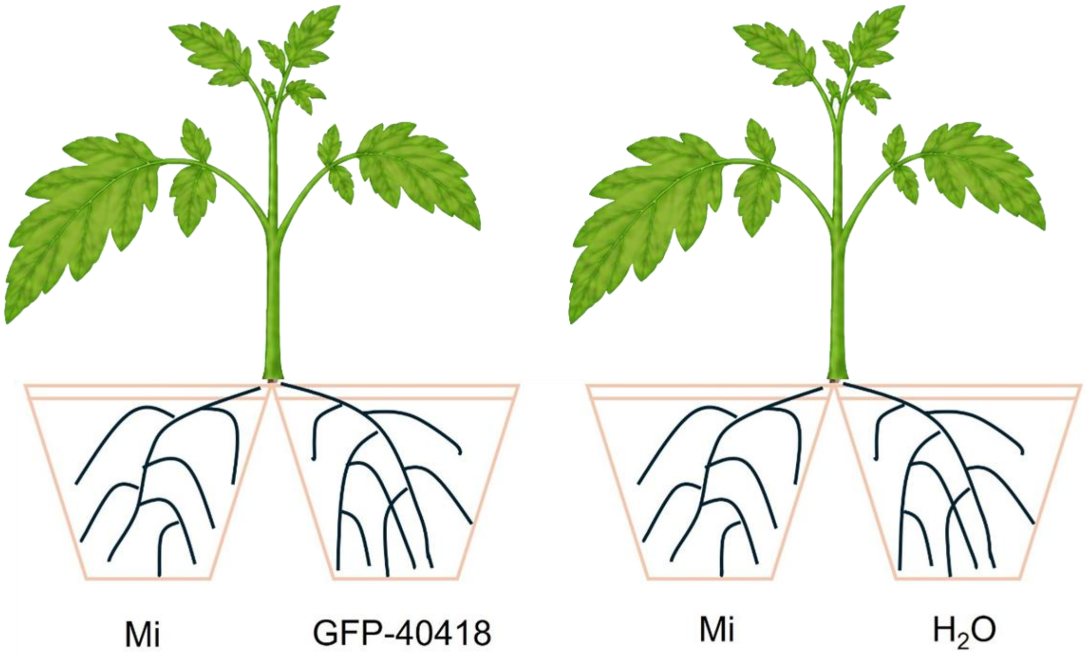

Figure S1. Schematic diagram of the split-root system

(A) Treatment group. (B) Control group.

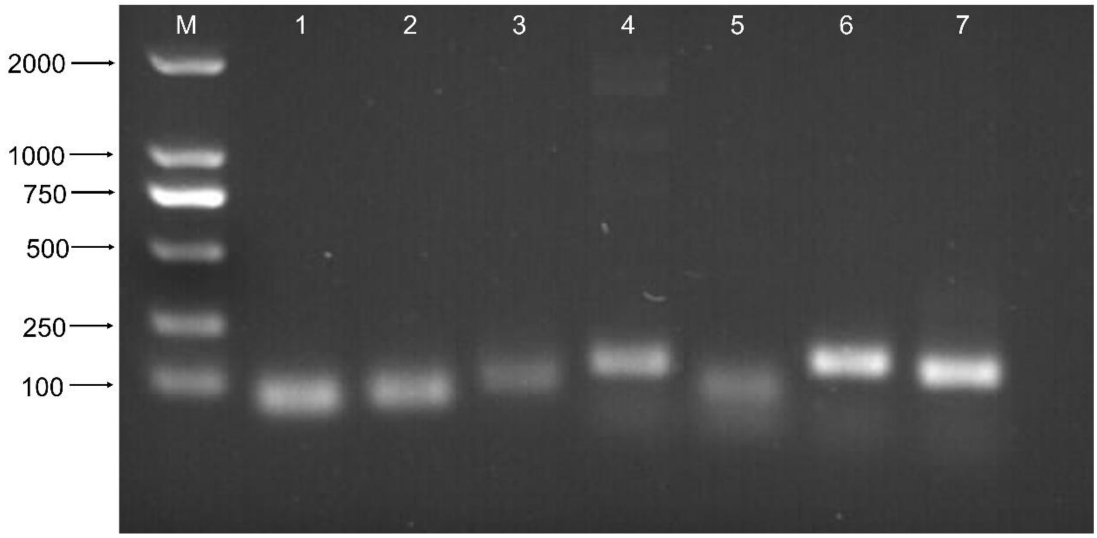

Figure S2. Gene-specific amplification profile

M: DL2000 Marker; Lanes 1 to 3: Lanes 1–3 correspond to *Actin*, *PR2*, *LOX*, *ETR1, Pal1*, *MYC2* and *ACO1*, respectively.
